# Structural similarity of genetically interacting proteins

**DOI:** 10.1186/1752-0509-2-69

**Published:** 2008-07-31

**Authors:** Oranit Dror, Dina Schneidman-Duhovny, Alexandra Shulman-Peleg, Ruth Nussinov, Haim J Wolfson, Roded Sharan

**Affiliations:** 1 School of Computer Science, Raymond and Beverly Sackler Faculty of Exact Sciences Tel Aviv University, Tel Aviv, 69978, Israel; 2 Basic Research Program, SAIC-Frederick, Inc. Center for Cancer Research Nanobiology Program, NCI, Frederick, MD 21702, USA; 3 Department of Human Genetics and Molecular Medicine, Sackler Faculty of Medicine, Tel Aviv University, Tel Aviv, 69978, Israel

## Abstract

**Background:**

The study of gene mutants and their interactions is fundamental to understanding gene function and backup mechanisms within the cell. The recent availability of large scale genetic interaction networks in yeast and worm allows the investigation of the biological mechanisms underlying these interactions at a global scale. To date, less than 2% of the known genetic interactions in yeast or worm can be accounted for by sequence similarity.

**Results:**

Here, we perform a genome-scale structural comparison among protein pairs in the two species. We show that significant fractions of genetic interactions involve structurally similar proteins, spanning 7–10% and 14% of all known interactions in yeast and worm, respectively. We identify several structural features that are predictive of genetic interactions and show their superiority over sequence-based features.

**Conclusion:**

Structural similarity is an important property that can explain and predict genetic interactions. According to the available data, the most abundant mechanism for genetic interactions among structurally similar proteins is a common interacting partner shared by two genetically interacting proteins.

## Background

Recent advance in systematic studies on the network level of several organisms provide new insights to the cellular complexity [1,2]. Systematic single gene deletion in yeast *S. Cerevisiae *revealed that fewer than 20% of all yeast genes are essential for growth on rich glucose medium [3]. This suggested that biological pathways are highly robust and lead to the development of high-throughput techniques for elucidation of the function and compensatory pathways of the non-essential genes [4,5].

Genetic interactions (GIs), in which two gene mutations have a combined effect not exhibited by either mutation alone, span overlapping functions and compensatory pathways. Recent developments of high-throughput techniques have enabled the large scale mapping of GIs [2]. The most common types of GIs, and the main focus of this work, are synthetic lethal and synthetic sick interactions, in which the combined mutation causes cell death or a growth defect. The analysis of these interaction types is crucial for identifying gene backups and compensatory pathways [6,7]. Synthetic genetic arrays (SGA), probing for these interaction types, have enabled the identification of 15,182 interactions in yeast *S. Cerevisiae *[8-10]. An additional GI network with 11,606 synthetic sick (aggravating) interactions in yeast have recently been defined by an epistatic miniarray profile (E-MAP) [11]. For the worm *C. Elegans*, 377 interactions have been identified by using RNA interference (RNAi) [12].

By their nature, GIs often relate functionally similar proteins. Indeed, Tong et al. report that 27% of the genetically interacting protein pairs in yeast have similar function [8]. In contrast, only 1–2% of the GIs share significant sequence similarity. Hence, the sequence similarity signal fails to capture most of the known GIs. Since protein structure is known to be more conserved than its sequence, we hypothesized that structural similarity may reveal GIs that cannot be detected at the sequence level.

Recent progress of the structural genomics project [13] has significantly increased the number of known protein structures. Currently, about 50% of the yeast and worm proteins have at least partial structural assignment using homologous proteins in other organisms [14]. This covers about 35% of the coding sequences in these genomes. Together with the development of large scale structural alignment tools [15], this allows to conduct a comprehensive comparison of protein structures among GIs.

Here, we performed a large-scale structural study of GIs. More than a million structural alignments were performed in order to estimate the prevalence of structurally similar GIs (St-GIs) in yeast and worm. We show that a significant fraction (7–14%) of the GIs in yeast and worm exhibit structural similarity and suggest a structure-based mechanism for such interactions. We also identify several structural features that are predictive of GIs. We combine these features within a logistic-regression-based framework for GI prediction and show their superiority over sequence-based features.

## Results 

### Structural similarity in GIs

To test the extent to which genetically interacting proteins display structural similarity, we carried out a large scale structural comparison analysis involving more than 10^6 ^alignments between protein domains whose encoding genes were tested for GIs in *S. Cerevisiae *and *C. Elegans *(see Methods). Briefly, bait (*query*) proteins used in large scale GI assays, and for which we had structure information [14], were compared to all non-essential (*target*) genes of the respective organism.

Notably, we found that significant numbers of GIs involved structurally similar proteins in both species (Table 1). In the yeast GI network defined by the synthetic genetic arrays (SGAs) of Tong *et al*. and Pan *et al*., 7.4% (298/4039) of the GIs with known structures involved structurally similar proteins (*p *< 1.2 *e *– 9 by a hypergeometric test). A similar percentage of structurally similar GI (*St-GI*) pairs (354/3527 = 10%) was found in the yeast GI network determined by Collins *et al*. In worm, 13.8% (11/80) of the GIs exhibited structural similarity (*p *< 0.04). These results show that St-GI pairs are abundant, and that structure information can explain a large percentage (7–14%) of the known GIs. The full lists of St-GIs in yeast and worm are provided as Additional files 1 and 2.

**Table 1:** Statistics of sequence and structural similarity among GIs and all gene pairs.

Organism	# GIs	# Gene pairs	Property	# Similar in GIs	# Similar in all gene pairs	*P*-value
Yeast	4039	103 × 2519	Structure	298 (7.4%)	13438 (5.2%)	1.3e-09
			Sequence	38 (0.9%)	650 (0.3%)	8.2e-12
Worm	80	9 × 1144	Structure	11 (13.8%)	792 (7.7%)	0.04
			Sequence	0	0	1

As molecular function can be inferred from protein structure, we hypothesized that St-GIs will tend to involve functionally similar proteins. Indeed, pairs with the same gene ontology (GO) annotation [16], were found to span significant fractions of St-GIs, even when compared to the set of all GIs, which is known to be enriched for functional similarity (Table 2). Expectedly, the most significant gain (35% vs. 10%) was observed for the Molecular function category, although significant gains were also observed for the Cellular component and Biological process categories.

**Table 2:** Statistics of functional similarity among GIs and St-GIs.

GO level	# GIs	# St-GIs	*P*-value
Cellular component	34%	47%	4.3e-07
Biological process	20%	40%	1e-17
Molecular function	10%	35%	7e-36

To compare the structural signal with the common sequence similarity measure, we tested the degree of sequence similarity among GIs using a BLAST *E*-value similarity threshold of 10^-6 ^(the corresponding p-value, corrected for multiple testing, is 0.05). As summarized in Table 1, only 0.9% of the yeast GIs exhibited sequence similarity (most of them displaying structural similarity as well), and none of the worm GIs did. In addition, using the SCOP classification we observed that only 49% of St-GIs are formed by proteins within the same SCOP superfamily and the remaining 51% of St-GIs are formed by proteins from different SCOP superfamilies. Moreover, about 25% of them stem from different folds.

**Figure 1:**
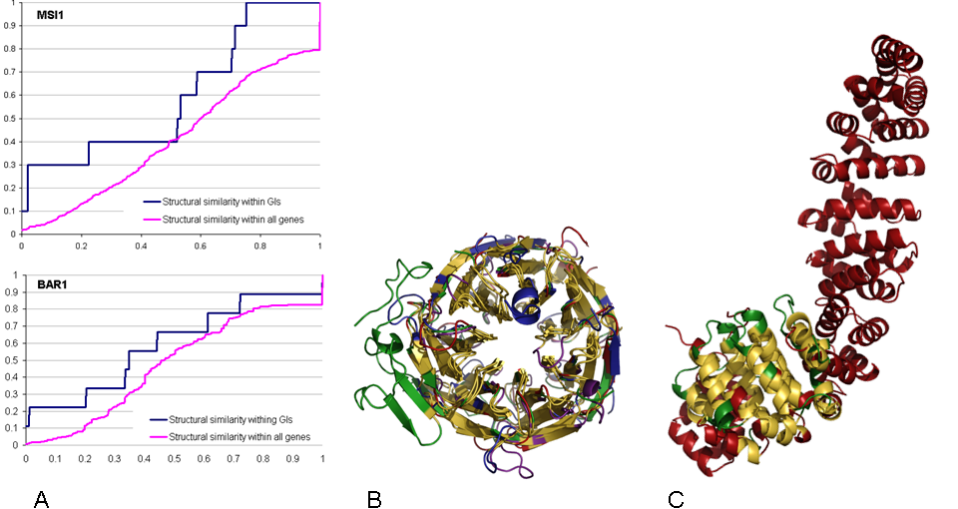
Examples of St-GIs. **(A) **Enrichment of structural similarity within GIs of two query genes, MSI1 and BAR-1. For each *p*-value (X-axis), plotted are the percentages of structurally similar proteins and of St-GIs with the query (Y-axis). **(B) **A multiple alignment of 4 protein domain structures: SCOP:87716 (red), SCOP:27660 (green), SCOP:107666 (blue), and SCOP:86078 (violet). These domains are involved in 3 GIs of the MSI1 query gene with genes HIR1, HIR2 and CTF4, respectively. The core of the alignment forms a beta-propeller (yellow). **(C) **An alignment of two protein domain structures: SCOP:19114 (red) and SCOP:19216 (green). The former domain is assigned to genes BAR-1 and HMP-2, while the latter is assigned to HGRS-1. The BAR-1 gene is known to interact with the two other genes. The core of the alignment forms a superhelix (yellow).

Below, we provide two examples of query genes whose sets of GIs are overrepresented with St-GIs (Figure 1A), and such that their St-GIs do not exhibit sequence similarity. The first example is the MSI1 query gene, which functions in chromatin assembly in yeast. This gene is involved in 29 GIs, 10 of which have structural information. Three out of these 10 interactions are between structurally similar proteins with a common role in transcriptional regulation. The domain structures assigned to the four proteins involved in these St-GIs belong to the SCOP WD40 repeat-like superfamily in the 7-bladed *β*-propeller fold [17]. Figure 1B presents their multiple alignment. The core consists of 196 residues with an average RMSD of 1.5Å and forms a *β*-propeller. The most structurally conserved region is along the propeller's axis at the pocket formed by the N-termini of the interior *β*-strands. This region is indeed known to be functionally important, as *β*-propellers have a significant preference for binding proteins and other ligands along their axis [18].

The second example is the BAR-1 query gene in worm. This gene participates in 18 GIs, 9 of which have structural information. Two of these interactions are St-GIs and involve three genes with a common role in embryonic development. The protein domain structure assigned to BAR-1 belongs to the SCOP Armadillo repeat superfamily of the *α – α *super-helix fold. One of the target genes was assigned to the same Armadillo repeat. The other gene was assigned to a structure from a different superfamily of the *α – α *super-helix fold. Nevertheless, 7 of its 8 helices are fully aligned with the BAR-1 structure (77 residues, average RMSD of 2.0 Å, Figure 1C). These helices form two repeat units that span part of the binding groove, where most protein interactions occur [18].

### Compactness of protein structures

In addition to structural similarity, which was found to be a prominent feature within GIs, we searched for other structural properties that characterize GIs. Specifically, we estimated the *compactness *of the protein structures by calculating their average density of amino acids (see Methods). Below we present two compactness attributes that distinguished GIs from non-GIs.

**Figure 2:**
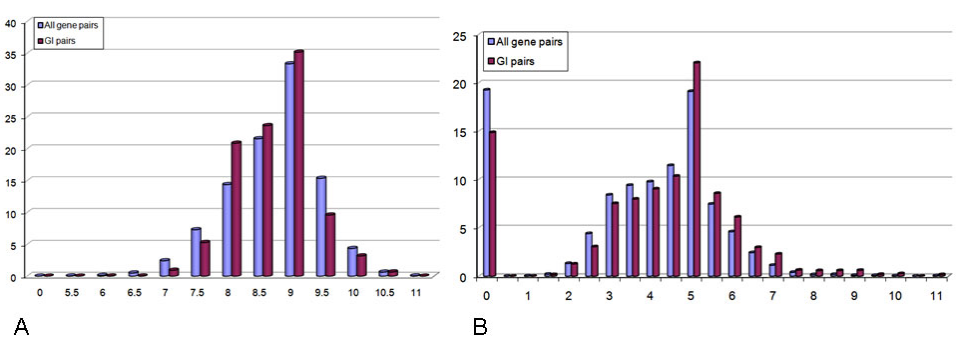
Statistics on compactness of GIs and all gene pairs. **(A) **A histogram of minimal query-target compactness values. **(B) **A histogram of structural alignment core compactness values.

The first attribute is the minimal domain compactness between the query and target proteins. We observed that medium domain compactness values (in the range 8.0–9.5, Figure 2) are indicative of GIs. This can be explained by the fact that functional promiscuity is sometimes linked to conformational diversity [19]. Medium compactness allows proteins to present a range of diverse, but still functional conformations. The second attribute is the compactness of the common substructure (*core*) of a structural alignment. Significant alignments between structurally similar proteins will usually have high core compactness which reflects the quality of the alignment. Consequently, we observed that high core compactness values (≥ 8.0) of significant structural alignments were indicative for GIs.

### GI prediction

We tested the predictive power of the identified structural features with respect to GIs. To this end, we implemented a logistic regression classifier combining the different features and tested its prediction performance (see Methods). Overall, we implemented several predictors based on sequence similarity, functional similarity (based on GO annotation) and structural similarity features. The latter structural features included: (i) the minimal compactness between the query and the target proteins; and (ii) the core compactness of the significant structural alignments.

**Figure 3:**
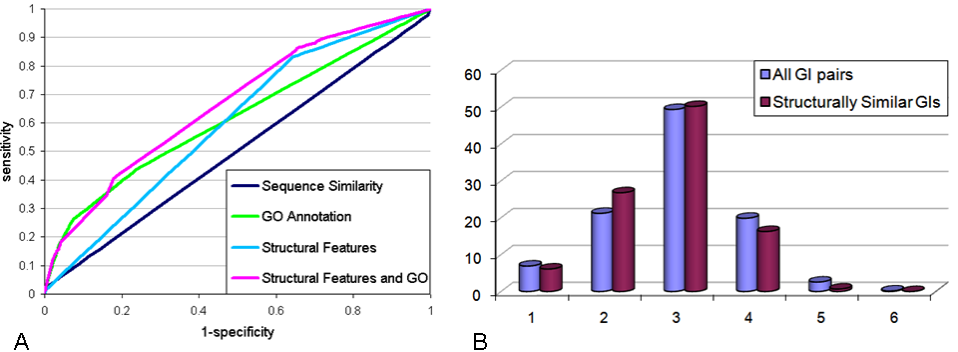
Properties of St-GIs. **(A) **ROC curves for GI predictors. **(B) **Distribution of PPI distances among GIs.

The performance of the predictors was evaluated using receiver operating characteristic (ROC) curves (see Methods). The area under such a curve is a standard way to assess the performance gain over a random predictor, which has an area of 0.5. Overall, sequence similarity performed poorly (ROC area of 0.5, Figure 3A) due to its low coverage of GIs (1–2% of the GIs). Nevertheless, if two proteins have a similar sequence we can predict with high confidence that they are in GI and sequence similarity is a very strong and specific predictor. On the other hand, for the remaining 98% of the GIs, without significant sequence similarity, we have to use alternative predictors. Notably, a predictor based on the structural features only attains a more significant area of 0.6 under the ROC curve. A third predictor based on functional annotations performs similarly (an area of 0.62). The combination of the functional and structural features provides the best predictor with an area of 0.67 under the curve. In conclusion, the structural features improve the prediction quality and outperform the sequence similarity feature.

### A structural mechanism for GIs

We suggest a possible mechanism for GIs among structurally similar proteins. In this mechanism, which we call *common friend*, genetically interacting proteins have a common interacting partner in a protein-protein interaction (PPI) network that binds to structurally similar domains of the two proteins. Such an interaction would mean that these proteins lie at distance two from each other in a PPI network. To support this hypothesized mechanism, we computed the distribution of pairwise distances among genetically interacting proteins in a PPI network and compared it to the distance distribution of St-GIs. We found that 26.8% of the St-GIs lie at distance 2 from one another, compared to 21.2% of all the GIs (*p-value *= 0.012) (Figure 3B).

Many examples of this mechanism revealed by our method are also supported by the biological literature. For instance, the two yeast genes for *α*-tubulin, TUB1 and TUB3, are in a St-GI and the two corresponding gene products have a common binding partner, *β*-tubulin (TUB2). Indeed, the absence of either TUB1 or TUB3 has an influence of the microtubule dynamics but it is not lethal [20]. Other examples include the St-GI between MYO2 and MYO4 with MYO1 as a 'common friend' [21] and the St-GI between SRS2 and RAD54 with their 'common friend' RAD51 [22].

## Discussion and conclusion

Here, we performed a genome-wide comparison of protein structures among GIs, and showed that significant fractions of genetic interactions involve structurally similar proteins. Moreover, we observed that structure similarity information is more predictive of GIs than sequence information. Although a large fraction of St-GIs is formed by functionally similar pairs, the protein function is not always dictated by its overall structure. For example, proteins with the same fold, like TIM barrels, can have multiple functions [23]. On the other hand, proteins with different folds, like subtilisin and trypsin, can share the same function. Consequently, we observed that a combination of structural and functional information within a logistic regression based predictor provides the best performance and is more indicative of GIs than either property by itself.

While our analysis has gained us several insights into the structural mechanism underlying GIs, several of its limitations should be acknowledged. First, current GI data sets contain very few false positives but high rates of false negatives (17–40%) [8]. Hence, our results might underestimate the utility of structural information in GI prediction. Second, structural information is far from complete. Structural information can be assigned to only 50% of yeast proteins. Additionally, even for these proteins, structures can be readily assigned only partially. Currently, many of the considered proteins do not have a complete structural coverage of their sequences and the average structural coverage is 45–60%. As a result, we might miss or underestimate similarities by concentrating on single domain alignments. In spite of this limitation, 11–20% of St-GIs were recognized to share more than one similar domain, suggesting a global structural similarity between the proteins. Last, proteins with different overall folds may perform similar functions and compensate each other due to binding site similarity. Large-scale investigation of such similarities is challenging and will be pursued in our future work.

## Methods

### Data acquisition

GI data were taken from BioGRID version 2.0.20 [24]. For yeast, two sets of query genes with known structures (for at least one of their domains) were used: 48 genes from [9,10] and 55 non-overlapping query genes from [8]. We excluded genes with ≤ 5 structurally covered GIs. The set of target genes consisted of 2,519 non-essential genes. These included all viable and lethal/viable genes in MIPS  with structural information for at least one of their domains. Overall, 4039 query-target pairs with structural coverage were reported to genetically interact. The *structural coverage *per query gene (that is, the total length of the protein subsequences for which domain structures have been assigned divided by the overall length of the protein sequence) was 53% on average. The protein structural coverage per target gene was 60% on average.

The worm data set consisted of 9 query genes and 1,144 target genes [12]. Overall, it spanned 377 interactions. On average, the protein structural coverage per gene was 44% and 54% for the query and target genes, respectively.

### Structural similarity computation

Genes were mapped to domain structures using the SUPERFAMILY database [14], which is based on the SCOP classification of protein domain structures [17].

Given a query gene *q *and a target gene *t*, we aligned each domain structure of *q *with each domain structure of *t *and computed the *p*-value of the resulting structural alignment. A gene pair (*q, t*) was considered to be *structurally similar *if at least one structural alignment between their domains attained a 0.05 significance level. All protein structural alignments were carried out by MultiProt [25] and MASS [26] with sequence order restriction.

### P-value computation

Given a structural alignment between a pair of protein domains, one of a query gene and one of a target gene, we estimated its significance by computing an empirical *p*-value of its core size with respect to a representative collection of pairwise structural alignments. For each protein domain of a query gene, we constructed a representative data set of alignments by computing all its pairwise alignments with a set of 1,538 protein domains representing all superfamilies of the seven true classes of SCOP [17]. Each alignment was assigned a size value, which denoted the minimum size of the participating domains. For a query domain, the *p*-value of a certain alignment with size *s *and core size *c *was defined as the fraction of alignments in the representative data set of size within 20% of *s *and core size exceeding *c*.

Since all alignments were performed using two methods, the final *p*-value for a domain pair was defined as the maximal *p*-value of the two. A query-target gene pair was considered *structurally similar *if the genes spanned a pair of domains with a final *p*-value ≤ 0.05.

### Compactness calculation

The compactness of a protein was calculated as the average number of neighbors of each residue. Two residues were considered neighboring if the distance between their corresponding *Cα *atoms was less than 8.0Å. The minimal domain compactness between query and target proteins was calculated between the domain pair with the most significant alignment. The core compactness was calculated as the average number of neighbors of the aligned residues.

### Sequence similarity computation

The gene sequence similarity was calculated by the Blastall software  applied to the complete yeast and worm genomes respectively. An *E*-value threshold of 10^-6 ^was used to ensure the significance of the alignments after taking into consideration the sizes of the genomes under study.

### GI prediction

We considered several features for GI prediction: (i) sequence similarity based, which included the BLAST *E*-value (-log transformed) of each pair; (ii) function similarity based, which included three binary variables indicating co-membership in the same GO SLIM class in each of the three GO levels (component, process and function); and (iii) structure similarity based, which included two binary variables indicating whether the minimal compactness and core compactness of the pair fall within predefined ranges (8.0–9.5 for the former and ≥ 8.0 for the latter).

Logistic regression based classifiers were constructed using R  (see Additional file 3). The classifiers were trained on the data set of Pan *et al*. [9,10]. The trained classifiers were then applied to the data set of Tong *et al*. [8] to produce a GI confidence estimate for each query-target pair. The prediction quality of a classifier was assessed by constructing a receiver operating characteristic (ROC) curve and computing the area under it. A ROC curve plots the true positive rate of the predictions (sensitivity) as a function of the true negative rate (1-specificity), while varying the prediction threshold. The sensitivity and specificity are defined as *TP/*(*TP *+ *FN*) and *TN/*(*TN *+ *FP*), respectively, where *TP *and *FP *are the number of correctly and incorrectly predicted GIs, and *TN *and *FN *are the number of non GIs that were predicted correctly and incorrectly.

## Authors' contributions

OD, DS and AS-P have performed the research and analyzed the data. All authors participated in the research design and manuscript preparation.

## Supplementary Material

Additional file 1 Structurally similar genetic interactions. The details of all yeast St-GIs recognized in the datasets of Tong *et al*., Pan *et al*. and Collins *et al*. Click here for file

Additional file 2 Structurally similar genetic interactions. The details of St-GIs observed in the worm network determined by Lehner *et al*. [12]. Click here for file

Additional file 3 Logistic regression details. The details of the constructed logistic regression predictors. Click here for file
