# Supplementary material for: Structural similarity of genetically interacting proteins
Source: BMC Syst Biol. 2008 Jul 31;2:69. doi: 10.1186/1752-0509-2-69 (PMC2525628; doi:10.1186/1752-0509-2-69)
Supplement: Additional file 3 — Logistic regression details. The details of the constructed logistic regression predictors. [file 1752-0509-2-69-S3.pdf]

Logistic Regression Details

```
Call:
glm(formula = trngFlag ~ eValue_seq, family = binomial, data = tpan,
    weights = w)

Deviance Residuals:
    Min       1Q   Median       3Q      Max
-0.4052  -0.1615  -0.1615  -0.1615   1.1977

Coefficients:
             Estimate Std. Error z value Pr(>|z|)
(Intercept) -0.004058   0.029069  -0.140   0.8890
eValue_seq   0.019056   0.010017   1.902   0.0571 .
---
Signif. codes:  0 `***' 0.001 `**' 0.01 `*' 0.05 `.' 0.1 ` ' 1

(Dispersion parameter for binomial family taken to be 1)

    Null deviance: 6590.4  on 128420  degrees of freedom
Residual deviance: 6585.4  on 128419  degrees of freedom
AIC: 3297.0
```

A

```
Call:
glm(formula = trngFlag ~ goMatch_C + goMatch_P + goMatch_F, family = binomial,
    data = tpan, weights = w)

Deviance Residuals:
    Min       1Q   Median       3Q      Max
-0.2713  -0.1594  -0.1467  -0.1467   1.2907

Coefficients:
            Estimate Std. Error z value Pr(>|z|)
(Intercept)  -0.2625     0.0364  -7.211 5.56e-13 ***
goMatch_C     0.2230     0.0675   3.303 0.000955 ***
goMatch_P     1.5698     0.1109  14.156  < 2e-16 ***
goMatch_F     0.2676     0.1332   2.008 0.044627 *
---
Signif. codes:  0 `***' 0.001 `**' 0.01 `*' 0.05 `.' 0.1 ` ' 1

(Dispersion parameter for binomial family taken to be 1)

    Null deviance: 6590.4  on 128420  degrees of freedom
Residual deviance: 6270.2  on 128417  degrees of freedom
AIC: 3172.4

Number of Fisher Scoring iterations: 4
```

B

```
Call:
glm(formula = trngFlag ~ coreCompB + minCompB, family = binomial,
    data = tpan, weights = w)

Deviance Residuals:
    Min       1Q   Median       3Q      Max
-0.2306  -0.1681  -0.1681  -0.1412   1.3346

Coefficients:
            Estimate Std. Error z value Pr(>|z|)
(Intercept) -0.36224    0.05815  -6.230 4.68e-10 ***
coreCompB    1.02005    0.27505   3.709 0.000208 ***
minCompB     0.47148    0.06711   7.025 2.14e-12 ***
---
Signif. codes:  0 `***' 0.001 `**' 0.01 `*' 0.05 `.' 0.1 ` ' 1

(Dispersion parameter for binomial family taken to be 1)

    Null deviance: 6590.4  on 128420  degrees of freedom
Residual deviance: 6529.1  on 128418  degrees of freedom
AIC: 3270.2
```

C

```
Call:
glm(formula = trngFlag ~ goMatch_C + goMatch_P + goMatch_F +
    coreCompB + minCompB, family = binomial, data = tpan, weights = w)

Deviance Residuals:
    Min       1Q   Median       3Q      Max
-0.3146  -0.1534  -0.1534  -0.1390   1.4634

Coefficients:
            Estimate Std. Error z value Pr(>|z|)
(Intercept) -0.65119    0.06506 -10.009  < 2e-16 ***
goMatch_C    0.24856    0.06804   3.653 0.000259 ***
goMatch_P    1.56663    0.11147  14.055  < 2e-16 ***
goMatch_F    0.22935    0.13611   1.685 0.091986 .
coreCompB    0.64903    0.29301   2.215 0.026755 *
minCompB     0.50690    0.06970   7.273 3.53e-13 ***
---
Signif. codes:  0 `***' 0.001 `**' 0.01 `*' 0.05 `.' 0.1 ` ' 1

(Dispersion parameter for binomial family taken to be 1)

    Null deviance: 6590.4  on 128420  degrees of freedom
Residual deviance: 6213.3  on 128415  degrees of freedom
AIC: 3142.5
```

D

Figure 1: The logistic regression predictors constructed by the R program. **(a)** The sequence similarity based predictor. **(b)** The GO annotation predictor. The binary variables $goMatch\_C$, $goMatch\_P$, and $goMatch\_F$ described the identity in the classification GO annotation levels: Component, Process and Function.**(c)** The predictor based on structural features: (1) the minimal compactness between the query and the target gene products (minCompB); (2) the structural alignment core compactness of the significant structural alignments (coreCompB). **(d)** The predictor based on the combination of the GO annotation with the structural features.
